# Does practicing hatha yoga satisfy recommendations for intensity of physical activity which improves and maintains health and cardiovascular fitness?

**DOI:** 10.1186/1472-6882-7-40

**Published:** 2007-11-30

**Authors:** Marshall Hagins, Wendy Moore, Andrew Rundle

**Affiliations:** 1 Division of Physical Therapy, Long Island University, One University Plaza, Brooklyn, NY 11201, USA; 2 Mailman School of Public Health, Columbia University, 622 W. 168th St. New York, NY 10032, USA

## Abstract

**Background:**

Little is known about the metabolic and heart rate responses to a typical hatha yoga session. The purposes of this study were 1) to determine whether a typical yoga practice using various postures meets the current recommendations for levels of physical activity required to improve and maintain health and cardiovascular fitness; 2) to determine the reliability of metabolic costs of yoga across sessions; 3) to compare the metabolic costs of yoga practice to those of treadmill walking.

**Methods:**

In this observational study, 20 intermediate-to-advanced level yoga practitioners, age 31.4 ± 8.3 years, performed an exercise routine inside a human respiratory chamber (indirect calorimeter) while wearing heart rate monitors. The exercise routine consisted of 30 minutes of sitting, 56 minutes of beginner-level hatha yoga administered by video, and 10 minutes of treadmill walking at 3.2 and 4.8 kph each. Measures were mean oxygen consumption (VO_2_), heart rate (HR), percentage predicted maximal heart rate (%MHR), metabolic equivalents (METs), and energy expenditure (kcal). Seven subjects repeated the protocol so that measurement reliability could be established.

**Results:**

Mean values across the entire yoga session for VO_2_, HR, %MHR, METs, and energy/min were 0.6 L/kg/min; 93.2 beats/min; 49.4%; 2.5; and 3.2 kcal/min; respectively. Results of the ICCs (2,1) for mean values across the entire yoga session for kcal, METs, and %MHR were 0.979 and 0.973, and 0.865, respectively.

**Conclusion:**

Metabolic costs of yoga averaged across the entire session represent low levels of physical activity, are similar to walking on a treadmill at 3.2 kph, and do not meet recommendations for levels of physical activity for improving or maintaining health or cardiovascular fitness. Yoga practice incorporating sun salutation postures exceeding the minimum bout of 10 minutes may contribute some portion of sufficiently intense physical activity to improve cardio-respiratory fitness in unfit or sedentary individuals. The measurement of energy expenditure across yoga sessions is highly reliable.

## Background

Physical activity conveys multiple well established health benefits including decreased rates of coronary artery disease [1-3], hypertension [4,5], non-insulin dependent diabetes mellitus [6], osteoporosis [7], colon cancer [8], anxiety and depression [9,10], as well as decreased risk of overall mortality [1]. Despite these clear benefits less than half of U.S. adults meet the current recommendations for physical activity [11].

The most recent recommendations from the American College of Sports Medicine (ACSM) and the American Heart Association (AHA) [12] suggest that to promote and maintain health all healthy adults aged 18–65 years old need moderate-intensity aerobic (endurance) activity for a minimum of 30 minutes on five days each week or vigorous-intensity activity for a minimum of 20 minutes on three days each week. These recommendations further suggest that combinations of moderate and vigorous exercise can be used to meet the requirements and that bouts of moderate-intensity exercise lasting a minimum of 10 minutes can be accumulated to achieve the 30 minute minimum. Recommendations for developing and maintaining cardiovascular fitness from the ACSM Position Stand recommend that exercise achieve 55–90% of the maximal heart rate, be performed 3–5 days per week, and last 20–60 minutes (continuous or intermittent) [13].

Yoga is an alternative form of physical activity which may assist in achieving recommended levels of physical activity for some individuals. Yoga is increasing in popularity [14-16] with a recent report suggesting that 15 million Americans have practiced yoga at least once in their lifetime [17]. Yoga classes are now offered at 75% of all U.S. health clubs [18], as well as at yoga studios and in private homes. Yoga may be attractive as an alternative to traditional aerobic and strength training programs because it requires little space, virtually no equipment, has limited harmful side effects [14,16,19,20] and, with its focus on relaxation, body awareness, and meditation provides a qualitatively different exercise experience which may be perceived as less strenuous and more pleasurable. Given these characteristics, yoga satisfies many of the conditions which have been shown to be strongly related to participation in physical activity, such as low perceived barrier to participation [21], being enjoyable [21], and having a low-to-moderate intensity [22].

Although yoga has received a considerable amount of study to date demonstrating significant cardio-respiratory [23-42], musculoskeletal [43,44] and metabolic health benefits [42], the degree to which the physical activity component of yoga may have contributed to these benefits has received virtually no attention. Surveying the English speaking literature available via Pubmed and CINAHL revealed eight studies examining the metabolic costs of yoga. Of these studies [45-52], only one [51] examined the variety of postures seen in a typical yoga class (standing, sitting, and lying). Additionally, all previous studies used measurement techniques that physically encumber the subject by requiring masks or mouthpieces to measure oxygen consumption. Such techniques may alter the performance of the yoga activities and therefore provide invalid estimates of metabolic costs.

Therefore, the purposes of this study were: 1) to determine whether an approximately one hour yoga session incorporating a variety of postures meets the current recommendations for physical activity sufficient to improve and maintain health [12] and cardiovascular fitness [13] as measured within a respiratory chamber (indirect calorimeter); 2) to determine the reliability with which the metabolic costs of yoga can be measured; 3) to compare the metabolic costs of yoga practice to the metabolic costs of steady state treadmill walking at two speeds (3.2 and 4.8 kph).

## Methods

### Subjects

Subjects were recruited via fliers posted at various yoga studios around New York, NY, USA, and on the Columbia University Health Sciences campus. Each participant was administered an eligibility questionnaire by telephone prior to enrolment. Exclusion criteria included: a) age < 18 years; b) < 1 year experience doing yoga; b) negative response to the question "are you comfortable doing Sun Salutation and basic yoga standing poses such as Triangle, Warriors I and II, and Tree?;" c) negative response to the question "are you able to do inversions (headstand, shoulder stand) with or without a wall?"; d) presence of any physical conditions or limitations that would prevent the individual from performing the tasks involved in the study. These exclusion criteria ensured that the participants would be able to engage in the yoga routine safely and that the measurements taken would not be influenced by inexperience.

### Measurement Instruments

The study took place at the Obesity Research Center at St. Luke's-Roosevelt Hospital, New York, N.Y., USA. After providing informed consent, each participant was outfitted with one Actiheart™ activity and heart rate logger (Mini-Mitter, OR, USA). The Actiheart™ is a small, lightweight device that was affixed to each participant's chest using standard EKG pads. It collected continuous heart rate (HR) data and produced data points at 1-minute intervals, reflecting the average heart rate for each minute.

Each participant performed all activities inside the respiratory chamber. The performance characteristics of the respiratory chamber have been described elsewhere [53,54]. Briefly, the system is an air-tight, temperature-controlled room in which rates of oxygen consumption (VO_2_) and carbon dioxide production (VCO_2_) are calculated as the difference between the composition of entering and exiting air. Analysis uses magnetopneumatic oxygen (Magnos 4G) and carbon dioxide (Magnos 3G) analyzers (Hartmann & Braun, Frankfurt, Germany), and the data is displayed and stored by the on-line computer system. The respiratory chamber provided the average values for VO_2_, VCO_2_, and energy expenditure (kcal/min) every two minutes. Energy expenditure was calculated via the respiratory chamber software using a modified Weir equation: EE = (3.94 × VO_2_) + (1.1 × (VCO_2_) [55].

Resting metabolic rate (RMR) for each participant was calculated by taking the mean energy expenditure during the last 20 minutes of the initial resting period. Each subsequent value for energy was divided by the RMR to determine metabolic equivalents (METs). To obtain the predicted maximum heart rate for each individual, the following formula was used: 208 – (0.7 × age) [56]. Heart rate data was multiplied by 100 and divided by the predicted maximum heart rate to determine the percentage of maximum predicted heart rate (%MHR) achieved by each individual for each 1-minute interval.

### Procedures

Subjects initially performed a 30 minute resting period during which they were seated and motionless except for the movements associated with reading (e.g., turning pages). Subjects then began a 56-minute beginner Ashtanga yoga DVD [57]. The yoga session began with 28 minutes of sun salutation poses (a standard series of moving poses found in many styles of yoga), followed by approximately 20 minutes of standing poses, and lastly, approximately 8 minutes of sitting and lying poses (relaxation). Table 1 describes the postures and time for each portion of the yoga session. For additional details see the commercially available "Ashtanga Yoga Beginner's Practice DVD [57]."

**Table 1:** Description of yoga practice

**Surya Namaskar (Sun Salutation)**	
*28 minutes*	

**Pose**	**Name**

Stand at Attention	*Samasthiti*
Hands above head	*Ekam*
Forward Bend	*Uttanasana*
Forward Bend/Head up	*Trini*
Down-dog	*Adhomukha Svanasana*
Four-legged staff pose	*Chaturanga Dandasana*
Up-dog	*Urdhvamukha Svanasana*
Chair	*Utkatasana*
Warrior I	*Virabhadrasana*

**Standing Poses**	

*20 minutes*	

Hands-to-feet	*Padahastasana*
Triangle	*Trikonasana*
Revolved triangle	*Parivrtta Trikonasana*
Side angle stretch	*Parivrtta Parsvakonasana*
Chest stretch	*Parsvottanasana*

**Sitting/Lying Poses**	

*8 minutes*	

Lotus	*Padmasana*
Corpse	*Savasana*

After completing the yoga practice, subjects walked on a treadmill at two different speeds in the following order: 3.2 kph (2 mph) and 4.8 kph (3 mph) for 10 minutes each. To determine across-session reliability, a convenience sample of seven participants returned between one week and one month later to perform an identical test protocol.

The first four minutes of HR and respiratory chamber data for each activity was discarded to allow each participant time to achieve a steady state of energy demand. Therefore, the data used for analysis included the final 20 minutes of the resting period, the final 52 minutes of the yoga session, and the final 6 minutes of each treadmill period.

### Statistical Analyses

SAS version 9.1 (SAS Institute Inc., Cary, NC, USA.) was used for all statistical analysis. Description of the means and standard deviations for all variables across the entire time periods of the resting, yoga, and treadmill activities were determined. Data from the seven subjects who repeated the measurement session were tested for reliability using Intra Class Correlation Coefficients (ICC) (2,1). Two separate one-sample t-tests were used to determine whether the mean METs across the entire yoga session and across the sun salutation portion of the yoga practice differed from the minimum values for METs (3.0) recommended by ACSM and AHA [12]. Additionally, two separate one-sample t-tests were used to determine whether the mean %MHR across the entire yoga session and across the sun salutation portion of the yoga practice differed from the minimum values of 55% MHR recommended by the ACSM Position Stand [13].

Three separate ANOVAs were used to compare the mean rate of energy expenditure (kcal), METs, and %MHR across the following activities: 1) rest; 2) entire yoga session; 3) treadmill walking at 3.2 kph; 4) treadmill walking at 4.8 kph. The sun salutation portion of the yoga session was not included in the first set of ANOVAs as the sun salutation portion and the entire yoga session are correlated and as such an analysis would have violated the assumptions of the ANOVA. Consequently, in order to compare the identical variables (kcal, METs, and %MHR) during the sun salutation portion of the yoga session a second set of three separate ANOVAs were performed on 1) rest; 2) sun salutation portion of the yoga session; 3) treadmill walking at 3.2 kph; 4) treadmill walking at 4.8 kph.

Because the order of the activities was not randomized and the increase in metabolism from the yoga activity may have influenced the metabolic rate during the ensuing treadmill activity, post hoc testing was performed to determine if the METs and %MHR during the final three minutes of the yoga program (during supine relaxation) had returned to values equal to those of the resting state.

## Results

**Figure 1:**
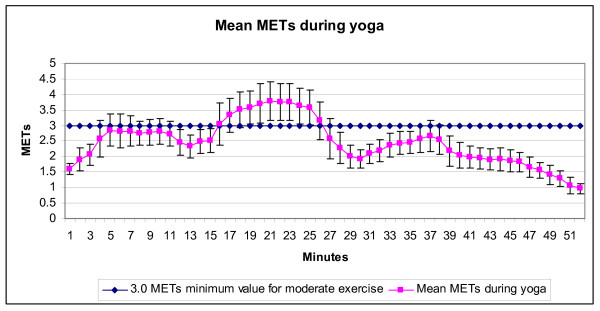
**Mean METs and standard deviation per minute during the yoga session (n = 20)**. Data from the first four minutes of the 56 minute yoga practice are not displayed. The postures used within each section of the yoga practice (see Table 1) varied in sequence and timing. Minutes 1–24 were sun salutation postures with postures changing every 3–8 seconds; minutes 25–44 were standing poses with postures changing every 10–48 seconds, and minutes 45–52 were sitting and lying postures lasting 3 minutes and 4.5 minutes respectively. Readers are referred to the yoga DVD for a complete description of the yoga session [57].

**Figure 2:**
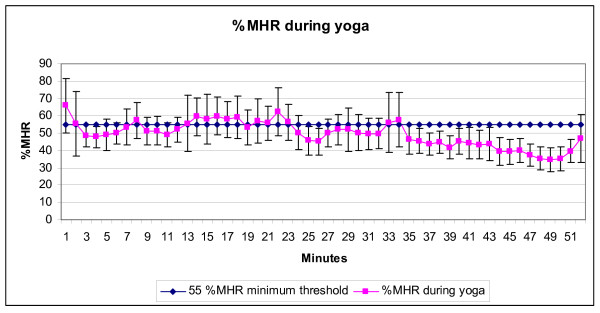
**Mean %MHR and standard deviation per minute during the yoga session (n = 18)**. Data from the first four minutes of the 56 minute yoga practice are not displayed. The postures used within each section of the yoga practice (see Table 1) varied in sequence and timing. Minutes 1–24 were sun salutation postures with postures changing every 3–8 seconds; minutes 25–44 were standing poses with postures changing every 10–48 seconds, and minutes 45–52 were sitting and lying postures lasting 3 minutes and 4.5 minutes respectively. Readers are referred to the yoga DVD for a complete description of the yoga session [57].

Twenty participants were recruited (18 female and 2 male). Results of the t-tests and ANOVAs were not different whether the two men were excluded from analysis or not, and therefore we report values from all 20 participants. The mean demographic values for the participants were: age 31.4, (± 8.3) years, height 165.2 (± 7.9) cm, weight 64.3 (± 9.0) kg, and BMI 23.58 (± 3.03). Means and standard deviations of HR, calories, VO_2_, METS, and %MHR relative to the entire yoga session, sun salutation postures, non-sun salutation postures, sitting/lying postures, and treadmill walking are described in Table 2. In a single subject, two values of energy expenditure during the final two minutes of the yoga session (during supine resting) were identified as outliers due to technical error (METs increased by a factor of ~5 between minutes 50 and 51 during supine resting (1.31 to 5.06 METs)). The outliers were replaced using the subject's value for the minute immediately prior to their occurrence (e.g., value at minute 50:1.31 METs). Figures 1 and 2 graphically describe the mean and SD of METs and %MHR, respectively, for each minute during the entire yoga session.

**Table 2:** Means and standard deviations for energy and heart rate parameters across all activities

**Task**	**Task**	Heart Rate (bpm)*	**Energy (kcal/min)**	**VO**_2_**(l/kg/min)**	**METs**	%MHR*
**Resting**	**(20 mins)**	71.0 (13.0)	1.2 (0.5)	0.3 (0.1)	1.0 (0.3)	38.2 (7.0)
**Yoga**	**Across entire Session (52 mins)**	93.2 (25.9)	3.2 (1.1)	0.6 (0.2)	2.5 (0.8)	49.4 (12.2)
	**Sun Salutation (24 mins)**	103.5 (25.2)	3.73 (1.01)	0.76 (0.21)	2.9 (0.77)	54.8 (11.8)
	**Non-Sun Salutation Standing Poses (20 mins)**	89.7 (23.9)	3.01 (0.810	0.61 (0.16)	2.34 (0.60)	47.3 (10.4)
	**Sitting/Lying poses (8 mins)**	72.5 (16.1)	1.93 (0.78)	0.40 (0.18)	1.5 (0.58)	38.7 (8.7)
**Treadmill**	**3.2 kph/Level (6 mins)**	97.8 (21.2)	3.1 (0.6)	0.7(0.1)	2.5(0.4)	50.7(8.0)
	**4.8 kph/Level (6 mins)**	110.3 (23.1)	4.2 (0.4)	0.9 (0.1)	3.3 (0.4)	58.1 (10.7)

*All subjects (n = 20) performed all activities. However, due to missing values, secondary to technical issues, data from only 18 subjects were included in %MHR calculations. MHR = Maximum predicted heart rate; METs = Metabolic equivalents;

Results of the ICCs (2,1) (n = 7) for mean values across the entire yoga session for metabolic cost (kcal), METs, and %MHR were 0.979, 0.973, and 0.865, respectively. The ICC value for %MHR *only *was based on 5 subjects, rather than 7, due to technical errors arising during data collection.

Results of the one-sample t-tests comparing mean METs and %MHR to recommended values are presented in Table 3. The mean METs across the entire yoga session were significantly less than the moderate level of physical activity reference value of 3.0 METs recommended for improving and maintaining health by the ACSM and AHA [12]. However, the mean METS during the sun salutation portion of the yoga session was not significantly different from the reference vale of 3.0 METs [12]. The mean %MHR during the entire yoga session was significantly less than the 55% of MHR described as a minimum threshold by the ACSM Position Stand [13] to achieve cardio-respiratory fitness. However, the mean %MHR during the sun salutation portion of the yoga session (24 minutes) was not significantly different from the 55% MHR recommended by the ACSM Position Stand [13].

**Table 3:** Results of one sample t-tests of mean METS and %MHR compared to recommended constant minimums

Variable	Comparison value	Difference between mean and comparison value	Standard Deviation	t statistic	P value
Mean %MHR* across entire yoga session (n = 18)	55% MHR [13]	-5.6	12.24	-14.38	< 0.0001
Mean %MHR* during sun salutation portion of yoga session (n = 18)	55% MHR [13]	-0.17	11.77	-0.30	0.7664
Mean METs across entire yoga session (n = 20)	3.0 METs [12]	-0.54	0.83	-20.81	< 0.0001
Mean METs during sun salutation portion of yoga session (n = 20)	3.0 METs [12]	-0.09	0.78	-2.80	< 0.0053

Note: *All values for MHR based on n = 18. Values for METs based on n = 20. MHR = Maximum predicted heart rate; METs = Metabolic equivalents;

Results of the three separate repeated measures ANOVAs examining mean rate of energy (kcal), METs, and %MHR across resting, the entire yoga session and two treadmill speeds found that all three variables differed significantly across the activities (p < 0.0001). All pair wise comparisons of the three variables were significantly different across all activities, with the exception that all three variables were not significantly different when comparing the entire yoga session to walking on a treadmill at 3.2 kph.

Results of the second set of three separate repeated measures ANOVAs (kcal, METs, and %MHR) in which the entire yoga session activity was replaced with the sun salutation activity found that all three variables differed significantly across the activities (p < 0.0001). All pair wise comparisons of the three variables were significantly different across all activities; with the exception that %MHR was not significantly different when comparing the sun salutations postures to walking on a treadmill at 4.8 kph.

By the end of the yoga session (during *Savasana*, resting supine posture) the mean METs had returned to 1, suggesting that the subjects had returned to the metabolic equivalent of a resting state. Post hoc statistical testing found that METs and %MHR during the final three minutes of the yoga session were not significantly different from mean values found during the initial rest period.

## Discussion

The current study found that the metabolic costs of yoga averaged across the entire session represent low levels of physical activity [58]. Mean values for kcal/min (3.2, ± 1.1), METs (2.5, ± 0.8), and %MHR (49.4, ± 12.2) were not significantly different from mean values during walking at 3.2 kph on a level treadmill. This level of physical activity was significantly lower than the recommendations for "moderate" levels of physical activity recommended by ACSM and AHA (3.0 METs) [12] and as recommended by the ACSM Position stand (55%MHR) [13].

Only four studies exist that may provide some comparison to the current study as they examined a variety of yoga postures which best represent a typical yoga class, similar to the current study [49-52]. Of these, one [52] provided insufficient description of the postures for valid comparison stating only that there were "six yoga positions." The remaining studies provided data on a series of well-described standing postures, however, the only study for comparison which included standing, sitting, and lying postures as in the current study is that of Clay *et al.*[51]. Clay *et al.*[51] studied a much shorter session (30 mins), a different selection of postures, younger subjects (difference of means: approximately 8 years), and used an indirect calorimeter requiring collection of gases from a mask or mouthpiece. These differences in methodology may explain why Clay *et al.*[51] found the mean caloric expenditure across the entire yoga session to be approximately 30% less than that found in the current study (2.23 vs 3.2 kcal/min).

Only two studies have provided %MHR during yoga for comparison to the current study [51,52]. The mean %MHR of 49.4% seen in this study appeared to be similar to that Clay *et al.*[51] which yielded a mean %MHR of 56.9%, and considerably less than that of Carroll et al. at 77%. However, the study by Carroll et al. represents standing postures only in a Vinyasa style (flowing movement) measured for 15 minutes, and therefore is unlikely to represent the mean %MHR across a typical yoga session.

It is important to recognize that the mean values across the entire yoga session described above represent a combination of standing, sitting, and lying postures and that substantial variation in metabolic costs occurs during the yoga session as can be seen in Figures 1 and 2. A substantial time period of the yoga practice (54%) was engaged in sun salutation postures which had metabolic values which were significantly higher than values for treadmill walking at 3.2 kph, were not significantly different than the recommended minimum values for moderate levels of exercise intensity (3.0 METs) [12], and were not significantly different from the minimum recommendations of the ACSM Position Stand for %MHR (55%, [13]) for cardiovascular fitness. Therefore, yoga practice with a portion of sun salutation postures exceeding the recommended minimum bout of 10 minutes [13] may contribute some portion of sufficiently intense physical activity to improve cardio-respiratory fitness. This being said, there are two important caveats regarding this suggestion. First, the criterion used for comparison in this study is the minimum value suggested by the ACSM Position Stand [13] (55%MHR). This value applies only to sedentary or unfit individuals, and therefore may not be a sufficiently high threshold for individuals who are currently fit (for more fit individuals the recommendations suggest a minimum of 65% MHR [13]). Second, ACSM Position Stand recommendations use %MHR as an indirect measure of cardio-respiratory demand based on the assumption of a relatively linear relationship between %MHR and oxygen consumption. However, there is evidence that during yoga, %MHR overestimates the amount of oxygen consumption used [49,51] and consequently %MHR may not be a sufficiently accurate measure of oxygen consumption during a yoga practice. Therefore, %MHR used in this study may be overestimating the intensity of the cardio-respiratory demand, and caution should be taken in consideration of this suggestion.

This study also demonstrated that individuals performing an identical yoga session (n = 7) on two occasions expend very similar amounts of energy (ICC = .979). This finding suggests that the use of mean metabolic values for quantifying energy cost of yoga across sessions is sufficiently consistent to provide a basis for valid estimates.

As is typical of most yoga classes, the yoga practice in this study ended with several minutes of supine relaxation (e.g., shavasana). The post hoc findings that METs and %MHR in the final three minutes of this relaxation were not significantly different from the mean value found in the initial resting period suggest that the increased metabolic demand of yoga was unlikely to have contributed to metabolic measures during the treadmill activity. In further support of this, the mean value (3.3) for the treadmill METs found in this study during the 4.8 kph was identical to the normative value suggested by Ainsworth for level treadmill walking at this same speed [58].

Placing the findings of the current study into a larger discussion of the potential for the physical activity component of yoga to provide health benefits requires consideration of the continuum of benefits that can be gained from increases in physical activity. The ACSM Position Stand [13] recommendations acknowledge that lower levels of physical activity than those recommended may provide health benefits for those who are quite unfit, particularly in the area of metabolic fitness [13]. Metabolic fitness [59,60] describes the ability of metabolic systems predictive of risk of cardiovascular disease (e.g., diabetes) to improve through intensities of physical activity which do not produce change in aspects of performance such as VO_2 _max. For example, in relation to obesity and Type II diabetes, overweight/obese individuals who increase physical activity can reduce insulin resistance even in the absence of concurrent weight loss [61-64]. Similarly among the non-obese, physical activity performed at levels insufficient to influence body mass or maximal oxygen uptake, can still improve insulin action [65]. The relatively low level of physical activity found in this study may serve to increase metabolic fitness despite being insufficient to create gains in cardio-respiratory fitness. This suggestion is in agreement with studies which have found yoga to improve indices associated with metabolic fitness such as insulin resistance [66,67] and lipid profiles [68,69].

There is a great deal of evidence that yoga or yoga based interventions reduce blood pressure and heart rate [23-42], and a recent systematic study suggests that over 85% of relevant studies published since 1970 demonstrate evidence that yoga reduces sympathetic tone, increases parasympathetic tone and improves cardiovagal function [42].

The mechanisms by which yoga conveys health benefits have received little study. However, given the findings of this study, as well as the study by Clay et al., it is unlikely that the well documented health effects across multiple systems are due solely to the low levels of physical activity within yoga practice. Yoga is a mind-body practice involving multiple integrated elements (e.g., conscious control of the breath, postures, meditation) [70]. The relative influence and interactions of these elements on health outcomes are unknown. In short, yoga may convey health benefits which are unrelated, or only partially related, to the physical activity component of yoga practice.

This study was limited in that it was a sample of convenience in which 90% of the subjects were women, and all were recruited from a private university and various urban yoga studios. Although, as mentioned previously, removing the two male participants from the data did not alter any of the results, it remains unclear whether a sample including more male participants would have altered the findings. All subjects were relatively young, healthy, and of normal weight and therefore the results may not be generalizable to persons suffering from adverse health conditions, those with very high or very low BMI, the elderly, or children under eighteen. The yoga DVD was chosen to represent the typical length, intensity, and variety of poses in a beginner yoga class, however, we have no information to determine the degree to which it is genuinely representative of yoga classes performed in the United States. Certainly, the results cannot be used to generalize to moderate or advanced level yoga classes. Also, as all subjects were moderate-to-advanced yoga practitioners, it is unclear how yoga experience may have influenced the findings in comparison to those who may be yoga novices. The lack of randomization of activities is a limitation in this study, suggesting that even though the study found that metabolic rate and %MHR returned to baseline after the yoga practice, possible influence upon the treadmill activities cannot be totally eliminated. Finally, in addition to the limitations described earlier relative to %MHR, we acknowledge that the true relative intensity of physical activity for each participant was not known as the VO_2 _max was not measured in this study, and consequently, more accurate data regarding each participant's percentage of VO_2 _max was not available.

## Conclusion

Metabolic costs of yoga averaged across the entire session represent low levels of physical activity, similar to walking on a treadmill at 3.2 kph, and do not meet recommendations for levels of physical activity for improving or maintaining health [12] or cardiovascular fitness [13]. Yoga practice with a portion of sun salutation postures exceeding the minimum bout of 10 minutes may contribute some portion of sufficiently intense physical activity to improve cardio-respiratory fitness in unfit or sedentary individuals. The measurement of energy expenditure across yoga sessions is highly reliable.

## Competing interests

The author(s) declare that they have no competing interests.

## Authors' contributions

MH and AR conceived of the study, participated in its design and coordination, and assisted with data analysis and drafting of the manuscript. WM performed data collection, assisted with organization and analysis of the data and drafting of the manuscript. All authors read and approved the final manuscript.

## Pre-publication history

The pre-publication history for this paper can be accessed here:


